# A morphometric study of the middle and lower cervical vertebral endplates and their components

**DOI:** 10.1097/MD.0000000000006296

**Published:** 2017-03-10

**Authors:** Hang Feng, Xiang-Yi Fang, Da-Geng Huang, Cheng-Cheng Yu, Hou-Kun Li, Song-Chuan Zhao, Chao-Yuan Ge, Ru-Hai Bai, Ding-Jun Hao

**Affiliations:** a Department of Spine Surgery, Honghui Hospital, Xi’an Jiaotong University Health Science Center; b Department of Public Health, Medical College, Xi’an Jiaotong University, Xi’an 710061, Shaanxi, China.

**Keywords:** artificial disc prostheses, cervical disc arthroplasty, cervical endplate, Chinese, morphology

## Abstract

Cervical disc arthroplasty is a common method of treating cervical degenerative disease. However, the footprints of most prosthesis dimensions are obtained from data of Caucasian individuals. Besides, there is a large discrepancy between footprints of currently available cervical disc prostheses and anatomic dimensions of cervical endplates. We aimed to detail the three-dimensional (3D) anatomic morphology of the subaxial cervical vertebral endplate, utilizing high-precision, high-resolution scanning equipment, and provide a theoretical basis for designing appropriate disc prostheses for Chinese patients.

A total of 138 cervical vertebral endplates were studied. Each endplate was digitized using a non-contact optical 3D range scanning system and then reconstructed to quantify diameters and surface area for the whole endplate and its components (central endplate and epiphyseal rim). The whole endplate and mid-plane concavity depth were measured.

There is marked morphologic asymmetry, in that the cranial endplate is more concave than the corresponding caudal endplate, with endplate concavity depths of 2.04 and 0.69 mm, respectively. For the caudal endplates, the endplate concavity apex locations were always located in the posterior portion (81.42%), while in cranial endplates relatively even. The central endplate was approximately 60% of the area of the whole endplate and the anterior section of the ring was the widest. From C3/4 down to C6/7 discs, the vertebral endplate gradually became more elliptical. Chinese cervical endplate anatomic sizes are generally smaller than that of Caucasians. Although Korean and Chinese individuals both belong to the Asian population subgroup, the majority of anatomic dimensions differ. Singaporean cervical endplate morphology is very similar to that of Chinese patients.

We performed a comprehensive and accurate quantitative description of the cervical endplate, which provide references to shape and profile an artificial cervical disc without sacrificing valuable bone stock. To design a device with footprint as large as possible to distribute the axial load, we suggest that additional attention should be paid to the marginal rim. It is essential to specifically design appropriate disc prosthesis for Chinese patients. To fit the morphologic and biomechanical variations, we also propose that the disc prostheses for different vertebral segments should be separately designed.

## 1 Introduction

Cervical disc replacement has emerged as an alternative surgical option to cervical arthrodesis, and has the potential to preserve motion at the operated level, provide biomechanical stability and global neck mobility, and reduce adjacent segment degeneration.^[1–4]^ However, there is a large discrepancy between footprints of currently available cervical disc prostheses and anatomic dimensions of cervical endplates.^[5]^ Furthermore, clinical outcomes have been reported for several complications related to size mismatch between the anatomic parameters of the cervical vertebrae and the footprint of the disc prostheses such as subsidence and heterotopic ossification.^[6–8]^

It is important to design an artificial disc that imitates the shape of endplates adjacent to a natural disc in all three dimensions. However, literature demonstrating accurate quantitative anatomic data on the vertebral endplate is sparse, especially with regards to the epiphyseal rim and the central endplate; the former is the strong and solid bony labrum, surrounding the outer rim of the vertebral body while the latter is the thin and porous central portion of the endplate.^[9,10]^ In addition, almost all prostheses are based on data obtained from Caucasian patients. Some studies have reported that Korean and Chinese Singaporean cervical vertebrae are smaller than Caucasian vertebrae.^[11,12]^ In fact, there is a large mismatch of available parameters of disc prostheses, as well as Chinese cervical anatomic data: 17.03% to 57.61% in the anterior–posterior diameter and 35.51% to 94.93% in the center of mediolateral diameter.^[13]^

We aimed to quantify the morphologic characteristics of the middle and lower cervical vertebral endplates and their components from Chinese cadaveric vertebral bones using digital measures, and to provide detailed reference data for the design and clinical use of the intervertebral devices.

## 2 Materials and methods

### 2.1 Samples

We obtained 19 Chinese cervical spine (C3–C7) specimens, which were spontaneously dried and stored at a constant temperature and humidity to prevent changes in shape or dimension. Intact vertebral endplates without pathologic deformation or broken parts were included; 11 endplates were excluded. Because of poorly scanned images, 3 endplates were also excluded, leaving a total number of 138 vertebral endplates (68 cranial and 70 caudal endplates). As the primary focus of this study was to quantify the three-dimensional (3D) morphology of the vertebral endplate and provide a theoretical basis to design disc prostheses, we specified the endplates as cranial or caudal with respect to the intervertebral disc. This study was approved by our institutional ethics committee.

### 2.2 Scanning and image processing

Each vertebral endplate was scanned using a non-contact optical 3D range scanning system (XTOM-micro I, Xi’an XinTuo 3D Optical Measurement Technology Co. Ltd., Xi’an, Shaanxi, China) to acquire surface geometric parameters. This high-speed and highly accurate flatbed scanner (precision 0.02 mm, 1628 × 1236 pixels, input time 3 seconds), can digitize the surface geometry of a targeted object. Before commencing data collection, the measuring instrument was calibrated and adjusted at an appropriate stand-off distance, according to the prescan images, to obtain the best image for the endplate. All scans were performed under uniform standard conditions.

After scanning, the endplate was converted into digital points called the point cloud. Then the point cloud was imported into Geomagic Studio (version 12; Geomagic Inc., Morrisville, NC) for further processing. Unneeded vertebral components in the acquired 3D virtual images, such as the posterior elements and osteophytes, were deleted, and only the vertebral endplate was preserved. Subsequently, the endplate point cloud was packaged into a stereolithography format file with reduced noise and spikes in Geomagic Studio. In brief, the vertebral endplate was scanned into a total of 45,000 to 70,000 digital points. Finally, the file was imported into reverse engineering software (Catia, version V5R20; Dassault System, Paris, France) and a 3D image was generated for each endplate to measure its surface geometry.

### 2.3 Measurement of endplate geometry

#### 2.3.1 Linear parameters (mm)

**Figure 1:**
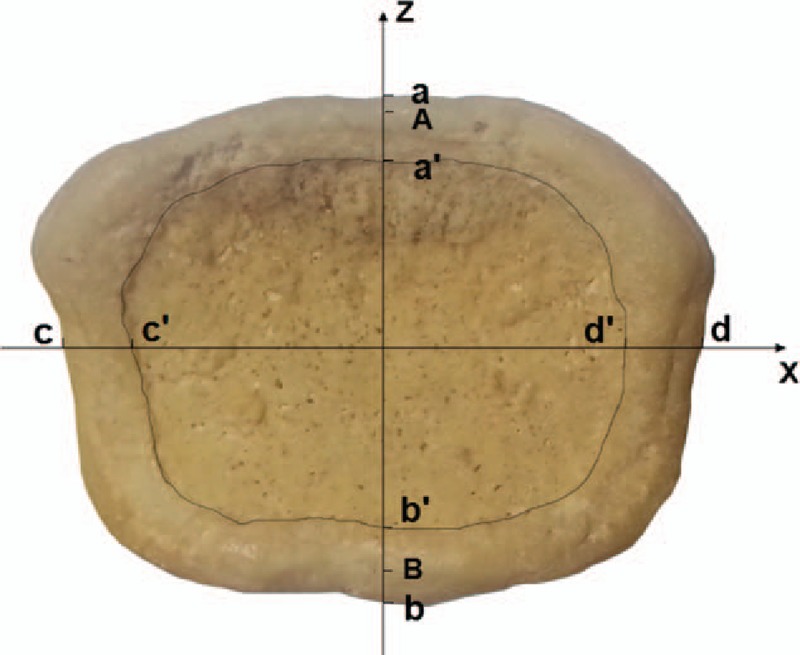
A image of vertebral cranial endplate. The vertebral endplate consists of the solid epiphyseal rim and the porous central endplate, that were separated manually (dash line) to measure their lines and surface areas. The axial reference plane was defined using 3 points from the epiphyseal rim (the left and right endpoints of the endplate trailing edge and the intersection A of the tangent line with the anterior median endplate rim). Point B was the intersection of the tangent line with the posterior median endplate rim, and line AP was used to calculate the mid-sagittal concavity apex depth and location. Diameter measurements were acquired from *z* axis (the mid-coronal plane) and *x* axis (the mid-sagittal plane). Line ab and cd represented anteroposterior diameter (APD) and transverse diameters (TD), respectively. Line a′b′ and c′d′ represented the AP and TD of the central endplate. For the epiphyseal rim, line aa′ and bb′ represented the anterior and posterior widths and the mean of the cc′ and dd′ represented the lateral width.

For both the whole and central endplates, the anteroposterior diameter (APD) was measured from the mid-sagittal plane. The transverse diameter (TD) was defined as the maximal distance in the mid-coronal plane; however, for the caudal endplate, the TD was defined as the distance between the furthest anterior points of the bilateral uncovertebral joints. Besides, to distinguish the uncovertebral joint from the caudal endplate in the reconstructed 3D image, a best-fit plane was defined through 4 scattered points (the anteriormost points and the posteriormost points of the bilateral uncinate processes) using the least squared method. And then, the intersection curves of the best-fit plane and the endplate surface were considered the boundary between the uncovertebral joint and the caudal endplate. For the epiphyseal rim, the anterior and posterior widths were measured from the mid-sagittal plane, and the lateral width was measured as the mean of the lateral right and left ring width acquired in the coronal plane (Fig. 1). Because of the uncovertebra, the lateral width in the caudal endplate was not measured. The endplate circularity, defined as the ratio of the TD to the APD, was calculated. It reflects the axial shape of the endplate, without taking concavity into account; the circularity of a circle is 1.^[14]^

#### 2.3.2 Surface area parameters (mm^2^)

Catia software (Catia, version V5R20; Dassault System, Paris, France) can directly measure the surface area of interest. The central portion of the endplate and the epiphyseal rim were separated by manually segmenting the regions of interest along the boundary between them in the 3D images of the endplate. Corresponding surface area measurements (measurements of the area of the 3D surface) were acquired for the whole endplate, the central endplate, and the epiphyseal rim (Fig. 1).

#### 2.3.3 Concavity parameters (mm or %)

We measured, respectively, the whole endplate concavity depth (ECD) and the mid-sagittal plane concavity depth (SCD). Before measurements were performed, the axial reference plane was defined using 3 points from the epiphyseal rim (the left and right endpoints of the endplate trailing edge and the intersection A of the tangent line with the anterior median endplate rim, Fig. 1). The perpendicular distance between the reference plane and the most concave point on the endplate surface was termed ECD. In the same way, the perpendicular distance between line AB (B was the intersection of the tangent line with the posterior median endplate rim, Fig. 1) and the concavity point in the mid-sagittal plane was termed SCD. In addition, we calculated two concavity apex locations, respectively. The endplate concavity apex location (ECL), was represented as the distribution of the most concave point in the axial reference plane. The reference plane was divided into 4 portions: the left-anterior portion, the right-anterior portion, the left-posterior portion, and the right-posterior portion. Then, we could determine the endplate concavity apex distribution according to its projective point in the reference plane. The projective point of the concavity apex in the mid-sagittal plane in line AB determined the relative location of the sagittal concavity apex (SCL), represented as the length of point A and the projective point divided by the length of AB.

### 2.4 Statistical analysis

Descriptive statistics (means and standard deviations) were obtained for quantitative variables. *T* tests and analysis of variance were employed for data analysis. The reliability, validity, and precision of the scanner for recording surface geometry are well established.^[14–16]^ To examine intra-rater reliability, 8 endplate samples were randomly selected after 2 weeks, using intra-class correlation coefficients. This digital measurement was highly reliable (all intra-class correlation coefficients >0.82). Statistical analyses were performed with SPSS software (version 18.0, SPSS Inc., Chicago, IL). Figures were created by GraphPad Prism 5 (GraphPad Software Inc., San Diego, CA).

## 3 Results

### 3.1 Linear parameters and surface area parameters

For the whole endplate, the mean size was 15.53 × 17.5 mm (APD × TD) for the cranial endplate and 15.07 × 17.86 mm for the caudal endplate. The average area of the endplate surface was 260.56 mm^2^ for the cranial endplate and 245.58 mm^2^ for the caudal endplate. The means and standard errors for the measured linear parameters and surface area parameters were shown in Table 1.

**Table 1:**
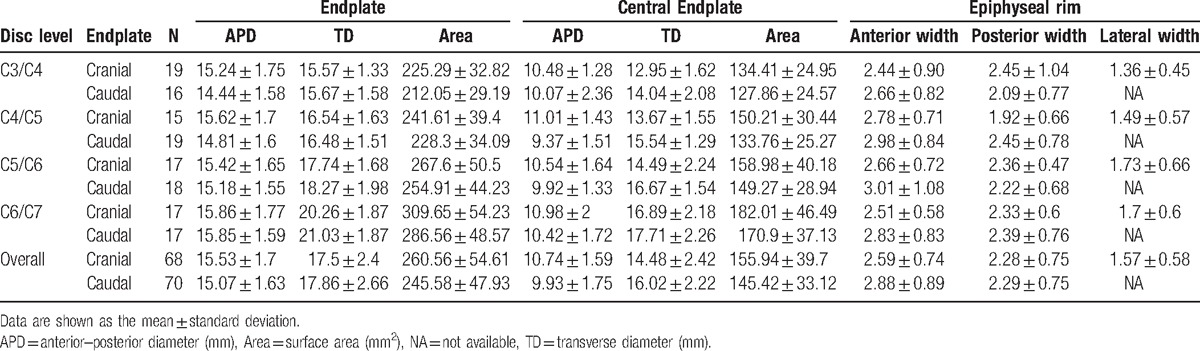
Linear and surface area parameters of the middle and lower cervical endplate.

**Figure 2:**
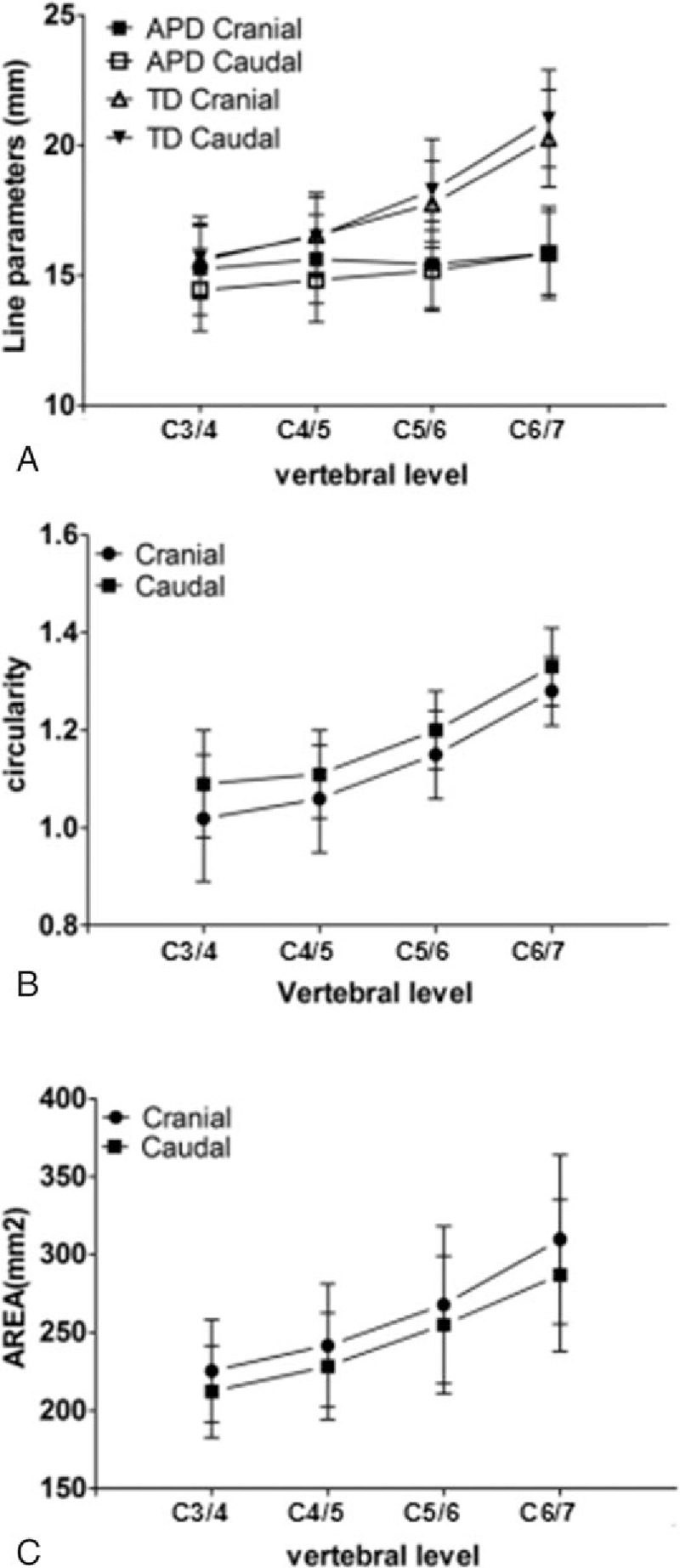
(A) Linear dimensions of the endplate of the cervical vertebral endplate; (B) circularity (TD/APD); (C) endplate surface area. APD = the anteroposterior diameter; TD = the transverse diameter.

The APDs were relatively constant in the cranial and caudal endplates (*P* > 0.05). The TD increased drastically, and were always greater than the corresponding APD (*P* < 0.01). For the cranial endplate TD, a significant increase was observed at the cranial of C6/C7 disc (*P* < 0.01); the TD of the caudal endplate had similar trend, with a significant increase observed at the caudal C5–C6 and C6–C7 discs (*P* < 0.01, Fig. 2A). Therefore, the circularity of the endplate increased gradually from 1.02 to 1.33; in other words, the endplates became more elliptical (*P* < 0.01, Fig. 2B). The total surface area increased from C3 to C7; for the cranial endplate, the areas rose significantly at the cranial C6–C7 level, and for the caudal endplate at the caudal C5–C6 and C6–C7 levels (*P* < 0.05). We further compared surface areas between the cranial and caudal endplates adjacent to the same disc level by level. For cervical intervertebral discs between C3–C4 and C6–C7, the surface area of the cranial endplate was greater than that of the corresponding caudal endplate (*P* < 0.05 for all discs, Fig. 2C).

Regarding the central endplate, for the cranial endplate, the APD was 10.74 ± 1.59 mm and the TD was 14.48 ± 2.42 mm, accounting for 69.16% and 82. 4%, respectively; the caudal endplate APD was 9.93 ± 1.75 mm, occupying 65.89% of the whole endplate. The average area of the central endplate was 155.94 mm^2^ for the cranial endplate and 145.42 mm^2^ for the caudal endplate, contributing 59.85% and 59.22% to the endplate area, serially. For the epiphyseal rim, the anterior width/diameter of the epiphyseal rim (AD) in the cranial and caudal endplates, with a similar trend for both posterior width/diameter of the epiphyseal rim (PD) and TD (*P* < 0.05). The AD was significantly wider than the PD in the cranial and caudal endplates (*P* < 0.01); besides, the narrowest rim in the cranial endplate was in the lateral region (*P* < 0.001).

### 3.2 Endplate concavity parameters

As shown in Table 2, the ECD was 2.04 ± 0.49 mm for the cranial endplate and 0.69 ± 0.29 mm for the caudal endplate; the SCD was 2.00 ± 0.50 mm and 0.65 ± 0.29 mm, respectively—significantly smaller than the ECD (*P* < 0.001). Overall, the endplates cranial to the disc had greater concavity depth than the caudal endplates, and the same for the ECD. Then, we further compared the ECD between the cranial and caudal endplates adjacent to the same cervical disc, level by level. We found that the cranial ECD was greater than that of their counterparts (*P* < 0.001 for all discs), suggesting a more even geometry on the caudal side of the cervical vertebral body (Table 2).

**Table 2:**
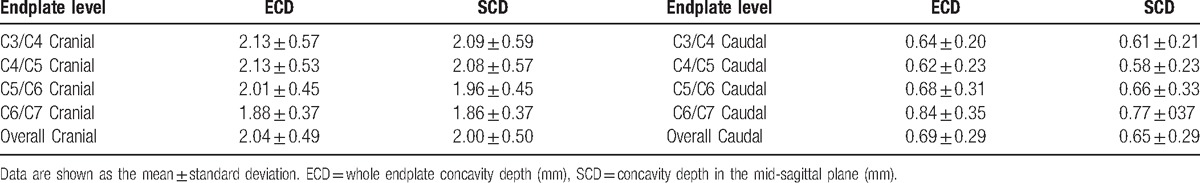
Whole endplate concavity depth, and concavity depth in the mid-sagittal plane.

For the 70 caudal endplates, the ECL had a much more observable difference. Thirty-two were in the right-posterior portion, accounting for 45.71%, and 25% in the left-posterior portion, and only 6 and 7 in the right-anterior portion and left-anterior portion, respectively. In general, the ECL was always located in the posterior portion (81.42%), with no significant differences between the left portion (55.71%) and right portion. However, the ECL in 68 cranial endplates was relatively even, with a minimum of 11 in the left-anterior portion and a maximum of 23 in the right-anterior portion (Table 3). We also calculated the concavity apex location in the mid-sagittal plane, namely the SCL (Table 4). In general, for the caudal endplate, the SCL was usually located in the posterior portion, and ranged from 55.31% to 69.56%, however, for the cranial endplate, most SCL distributed near the middle on the surface, and ranged from 48.38% to 51.41%. In addition, we further compared the difference of the ECL and the SCL in the anterior and posterior portions; there was no significant difference between the two (all *P* > 0.05), meaning that we could infer ECL by measuring SCL by CT or x-ray.

**Table 3:**
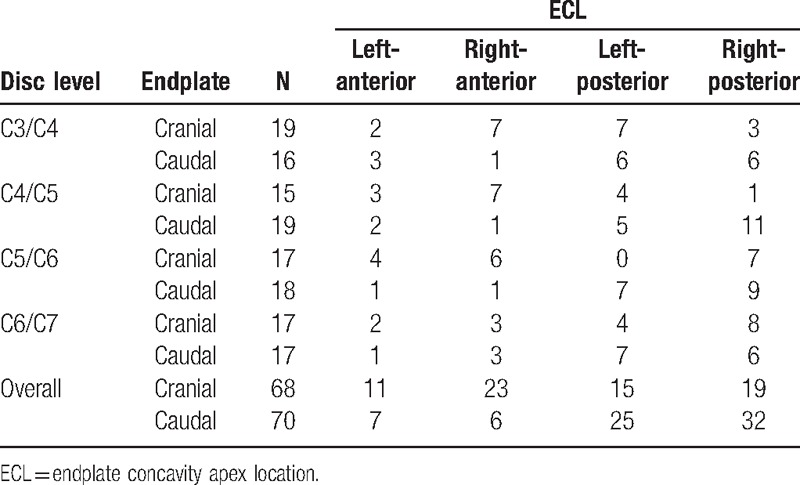
Location of endplate concavity apex in the transverse plane.

**Table 4:**
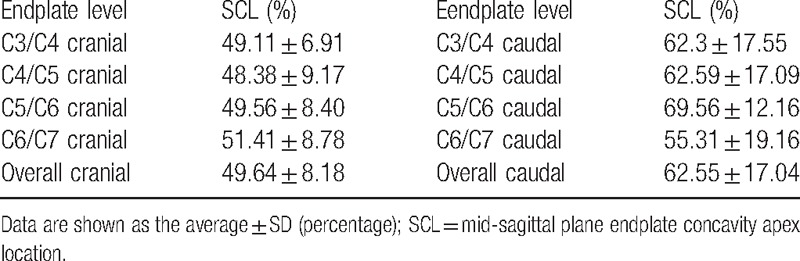
Location of endplate concavity apex in the mid-sagittal plane.

## 4 Discussion

There is marked morphological asymmetry between the two adjacent endplates of a cervical intervertebral disc: the cranial endplate was more concave than the corresponding caudal endplate, with a difference of 1.39 mm. However, almost all artificial disc prostheses used now have a relatively flat rather than an arcuate surface.^[17]^ To accommodate the implant, the endplate must often be polished to a flat plane, which undermines the integrity of the endplate and reduces its ability to withstand pressure. Cheng et al,^[18]^ reported that there was on average a 47% loss of stiffness when 1 mm of endplate was removed and 54% loss when 2 mm was removed. Lowe et al,^[19]^ demonstrated that complete removal of the endplate resulted in a decrease of nearly 39% in compressive strength. de Beer and Scheffer,^[20]^ performed a bio-mechanical investigation using 2 different endplate designs. The results revealed that during nondestructive tests, average percent contact area measured was 45.27% for conformal implants matching bone interface geometry, and 10.49% for implants with flat endplate geometries. Moreover, the conformal implants achieved higher failure loads during destructive compression tests. Yu et al,^[17]^ builded a new cervical artificial disc prosthesis based on the physiological curvature of the endplate, and compared the bio-mechanical differences with the Prestige LP prosthesis using a finite element model. They reported that the stress on the new artificial disc was significantly less than that in the Prestige LP prosthesis.

Due to the lack of the support from the stronger peripheral endplate epiphyseal rim, the undersized prosthesis is mainly located on the central endplate, which is thin and porous. Some studies have revealed that device subsidence is mainly caused by the limited contact area between prosthesis and endplate, which leads to point loading at the prosthesis-endplate interface.^[21,22]^ Hence, the prostheses should have a footprint as large as possible to dissipate the load evenly, rather than in concentrated areas.^[23]^ Although a few studies have reported quantified data regarding the endplate geometry,^[11,12,24–26]^ data concerning the quantitative relation between the compact rim and the much less resistant cancellous tissue in the central area is very scarce, which can help to define the exact contact area of disc prosthesis and endplate. Different from previous studies, the present study conveniently and accurately measured the endplates by introducing an optical 3D range scanning system and a reverse engineering software, which allowed not only the quantification of concavity, but also separate measurements of the central endplate and the epiphyseal rim. The findings that the area of the central endplate was about 60% of the entire endplate and the anterior section of the ring was the widest (2.44–3.01 mm) may provide a reference for designing artificial disc to obtain support from the epiphyseal rim of the endplate.

Although currently available disc prostheses have various footprint sizes, the shape of the sort of prosthesis utilized at different vertebral segments is almost the same.^[27]^ However, in the present study, we found that the vertebral endplate gradually changed into a more oval shape from C3–C4 to the C6–C7 disc, especially at the C6/C7 disc. In addition, the endplate surface area increased from C3 to C7, and the area of the cranial endplate was greater than that of the corresponding caudal endplate. Furthermore, the variation in the endplate shape could alter the biomechanical properties of the implant.^[11]^ Penzkofer et al,^[28]^ reported a device with an anatomical shape can provide mechanical advantages under imperfect alignment and may thus reduce secondary dislocation and the loss of correction. Therefore, it is necessary that the morphologic variations between different vertebral segments should be taken into consideration when designing disc prostheses.

**Figure 3:**
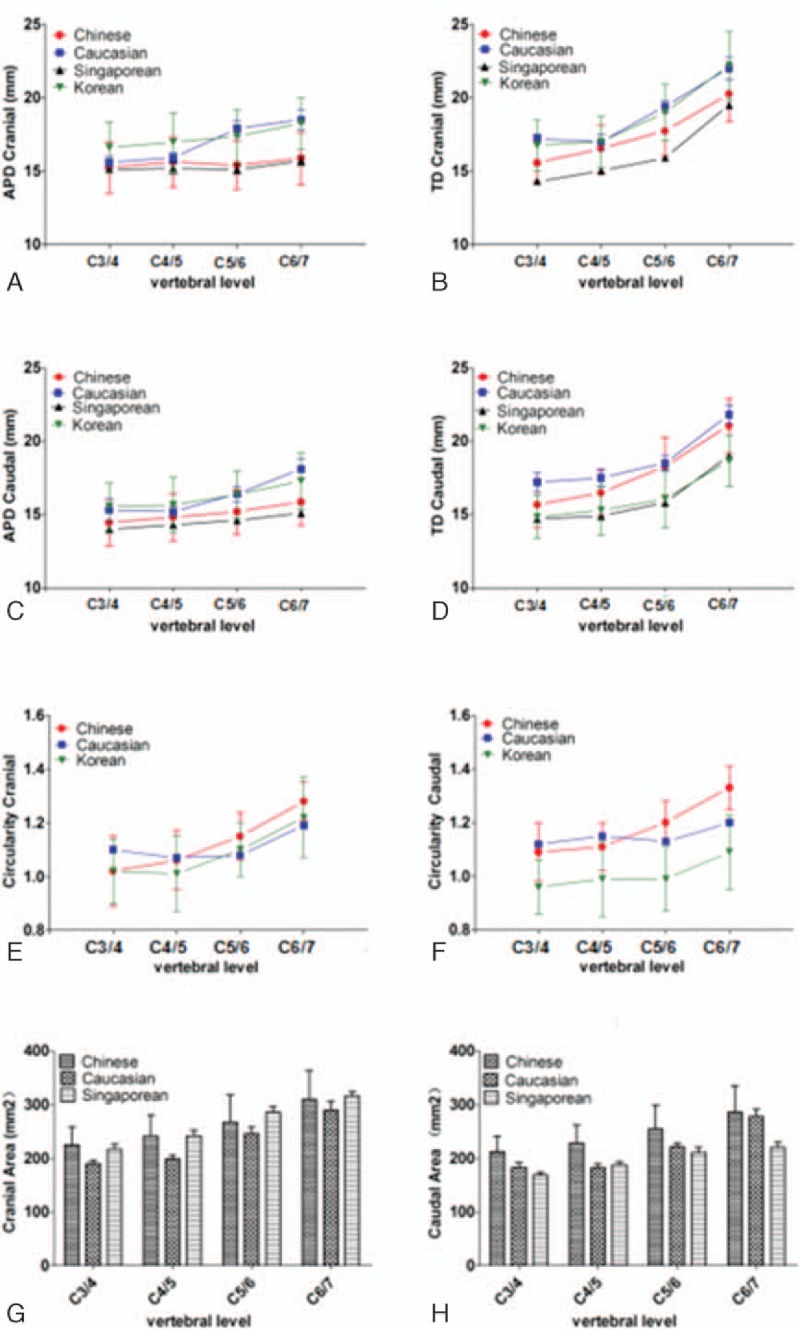
Comparison of the linear dimensions of the present study with Caucasian,^[24]^ Singaporean,^[12]^ and Korean.^[11]^ APD, the anteroposterior diameter (A and C), TD, the transverse diameter (B and D). Comparison of the circularity values of the present study with Caucasian and Korean. Circularity, TD/APD (E and F). Comparison of the surface area of the present study with Caucasian and Singaporean (G and H).

There are several excellent previous studies, whose parameters are compared with the present study. The linear parameters (APD and TD) obtained from Chinese, Caucasian,^[24]^ Singaporean,^[12]^ and Korean^[11]^ subjects are compared in Fig. 3A–D. The differences of circularity (TD/APD ratio) between Chinese, Caucasians, and Korean are shown in Fig. 3E and F. The endplate surface area parameters obtained from Chinese, Caucasians, and Singaporeans are compared in Fig. 3G and H. For the linear parameters, in general, Chinese parameters agree well with that of Singaporeans, and both are smaller than that of Caucasians. Although with similar trends, the values obtained from Koreans are larger than that of Chinese, while the TD of the caudal endplate is shorter. In the present study, the circularities increase from C3 through C7, and are greater than that of Singaporeans and Koreans. At the C3/C4 level, the circularities of Caucasians are larger than that of Chinese; however, they are smaller below this level. In other words, the endplate of the cervical vertebrae of Chinese cadavers is observed to be transversely elongated. With regards to the endplate surface area, there are no significant differences between the Chinese and Singaporeans. Interestingly, although linear parameters of the Chinese and Singaporeans are smaller than that of Caucasians, the surface areas are greater. The reason may be that the measuring instrument was unable to precisely evaluate the surface area at that time (Caucasians were assessed in 1991). Moreover, the line and area parameters from the Chinese and Singaporeans are similar to each other, suggesting that genes play an important role in endplate morphology. These results underscore the need to collect and analyze specific data of the Chinese population, and design artificial cervical intervertebral discs for Chinese patients.

One of the limitations to our study is that we did not compare groups according to age and sex. Further investigations are necessary to obtain more accurate information from sex and age specific studies that include a larger normal population. Another concern is that the measurements were conducted using dried specimens, which may be different compared with fresh samples or in vivo.

## 5 Conclusion

This study accurately and comprehensively quantified the morphologic characteristics of the subaxial cervical vertebral endplates from cadaveric vertebral bones using digital measures. The results provide beneficial references to shape and profile an artificial cervical disc without sacrificing valuable bone stock. As well know, the footprint of the device should be as large as possible to distribute the axial load, we suggest that additional attention should be paid to the morphology and function of the marginal rim, which may bring added bonus. As Chinese cervical endplate anatomic sizes are generally smaller than that of Caucasians, it is essential to specifically design and manufacture appropriate disc prosthesis for Chinese patients. Moreover, to fit the morphologic and biomechanical variations, we also propose that the disc prostheses for different vertebral segments should be separately designed.

## Acknowledgments

The authors thank Xi’an Jiaotong University State Key Laboratory for manufacturing system engineering with the acquisition of the quantitative morphometric measurements.

They also thank Xi’an Jiaotong University, Department of Anatomy, who provided them with Chinese cervical specimens.
